# Supplementary material for: Influence of Natural Deep Eutectic Solvent Compositions on the Polyphenol Profile of Citrus aurantium By-Products from Yucatán, México
Source: Molecules. 2025 Nov 26;30(23):4551. doi: 10.3390/molecules30234551 (PMC12693696; doi:10.3390/molecules30234551)
Supplement: Supplementary file 1 [file molecules-30-04551-s001.zip › molecules-3979448-supplementary.pdf]

Supplementary Material

Influence of Natural Deep Eutectic Solvent Compositions on the Polyphenol Profile of *Citrus aurantium* By-Products from Yucatán, México

Joaquín Fernández-Cabal [1], Kevin Alejandro Avilés-Betanzos [1], Manuel Octavio Ramírez-Sucre [1], Juan Valerio Cauich-Rodríguez [2,*] and Ingrid Mayanin Rodríguez-Buenfil [1,*]

[1] Center for Research and Assistance in Technology and Design of the State of Jalisco, A.C Southeast Sub-Headquarters, Cadastral Record 31264, Km 5.5 Sierra Papacal–Chuburna Puerto Highway, Scientific and Technological Park of Yucatan, 97070, Yucatan, Mexico; jofernandez_al@ciatej.edu.mx (J.F.-C.); keaviles_al@ciatej.edu.mx (K.A.A.-B.); oramirez@ciatej.mx (M.O.R.-S.)

[2] Center for Scientific Research of Yucatán, Materials Unit, 43rd Street No. 130 × 32 and 34, Chuburna de Hidalgo. 97205, Yucatan, Mexico

* Correspondence: jvcr@cicy.mx (J.V.C.-R.); irodriguez@ciatej.mx (I.M.R.-B.)

Table S1. *C. C. aurantium* by-products TPC, TFC, TAA and Ax obtained using different NADESs and ultrasound-assisted extraction.

#EXP	Encoded values			Real values			Response variables* (mg/100 g DM)			
	X_1	X_2	X_3	HBD	MR (mol/mol)	AW (%)	TPC (mg GAE)	TFC (mg QE)	TAA	Ax*
**1**	-1	-1	-1	Fructose	1	50	3603.7 ± 52.9^{f}	1161.4 ± 17.5^{a}	1948.2 ± 38^{ef}	82.26 ± 1.14^{cd}
**2**	0	-1	-1	Glycerol	1	50	2062.7 ± 84^{bc}	1607.9 ± 46^{f}	1836.4 ± 24.2^{cd}	80.52 ± 0.29^{ab}
**3**	1	-1	-1	Glucose	1	50	1955.2 ± 29.9^{a}	1159.6 ± 73.6^{a}	1734.8 ± 24^{b}	81.57 ± 0.05^{bc}
**4**	-1	1	-1	Fructose	2	50	3266.3 ± 60^{e}	1385 ± 52.9^{de}	1964.9 ± 33.7^{f}	84.31 ± 1.15^{e}
**5**	0	1	-1	Glycerol	2	50	2088 ± 19.3^{c}	1555.3 ± 46^{f}	1635.3 ± 52.8^{a}	80.27 ± 0.25^{ab}
**6**	1	1	-1	Glucose	2	50	2128.8 ± 44.3^{c}	1313.9 ± 97.8^{cd}	1854.9 ± 54.7^{cd}	80.03 ± 0.14^{a}
**7**	-1	-1	1	Fructose	1	70	2643.3 ± 33.8^{d}	1253.6 ± 34.9^{bc}	1828.5 ± 27^{c}	83.10 ± 1.56^{de}
**8**	0	-1	1	Glycerol	1	70	1973.1 ± 53.5^{a}	1363.4 ± 46.1^{de}	1892.5 ± 19.9^{de}	79.99 ± 0.17^{a}
**9**	1	-1	1	Glucose	1	70	1993.4 ± 16.9^{ab}	1221.1 ± 17.7^{ab}	1719.5 ± 45.9^{b}	81.71 ± 0.7^{bcd}
**10**	-1	1	1	Fructose	2	70	2705 ± 35.3^{d}	1212.8 ± 46.2^{ab}	1863.1 ± 20.3^{cd}	82.85 ± 0.1^{cde}
**11**	0	1	1	Glycerol	2	70	1924.9 ± 69^{a}	1829.8 ± 17.8^{g}	1759 ± 51.7^{b}	80.31 ± 1.36^{ab}
**12**	1	1	1	Glucose	2	70	2124.8 ± 34.7^{c}	1443.8 ± 31.2^{e}	1741.3 ± 26.6^{b}	79.22 ± 1.36^{a}

Note: MR = Molar ratio of glucose per 1 mol of Choline chloride; AW = Added water to NADES; HBD = Hydrogen Bond Donor; TPC = Total polyphenol content; TFC = Total flavonoid content; TAA = Total ascorbic acid (expressed as ascorbic acid equivalent); Ax = Antioxidant capacity; *GAE = gallic acid equivalent; QE = quercetin equivalent; * % Inhibition of DPPH; DM = dry mass. Values are means ± SD (n = 3).

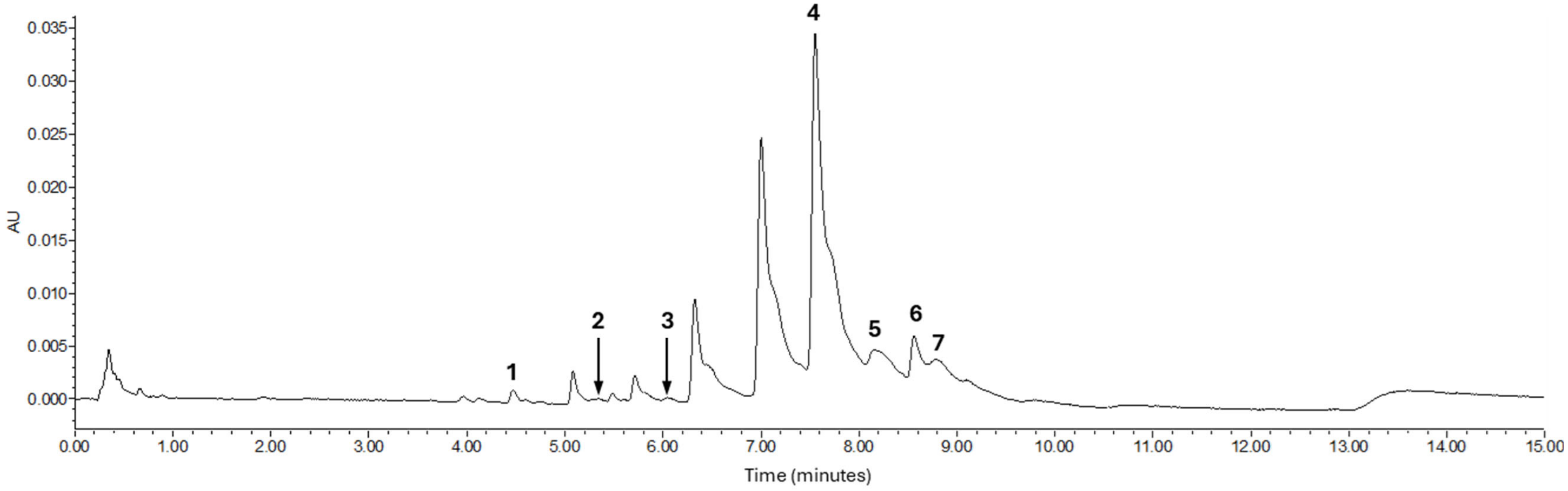

Figure S1: Chromatogram of C. aurantium extract obtained using NADESs formulated with glycerol as HBD, 1:1 MR and 70% AW. The numbers correspond to 1 = coumaric acid; 2 = cinnamic acid; 3 = rutin; 4 = hesperidin; 5 = quercetin + luteolin; 6 = neohesperidin and 7 = kaempferol.

Table S2. *C. aurantium* by-products polyphenol profile obtained using different NADESs and ultrasound-assisted extraction.

Exp*	Polyphenol profile (mg/ 100g DM)											
	**ClA**	**PrA**	**Ct**	**CoA**	**CiA**	**Rt**	**Q+L**	**Hp**	**Kp**	**Vn**	**Nhp**	**Ng**
**1**	42.51±0.18^{b}	ND	ND	6.39±0.01^{b}	25.30±1.96^{bcd}	92.49±4.35^{a}	244.08±1.01^{a}	1866.05±6.18^{e}	51.62±0.55^{a}	ND	10.42±0.72^{a}	ND
**2**	43.60±1.10^{cd}	ND	ND	12.57±0.01^{c}	24.15±0.84^{bc}	105.52±3.54^{cde}	721.32±23.05^{h}	1386.74±3.15^{cd}	240.59±9.24^{d}	8.90±0.13^{ab}	36.88±9.12^{c}	ND
**3**	44.14±1.05^{d}	ND	ND	12.94±0.23^{d}	24.63±1.66^{bcd}	96.59±0.33^{ab}	229.69±0.98^{a}	1339.31±3.42^{b}	213.01±9.24^{bc}	8.78±0.03^{ab}	25.20±1.93^{b}	25.07±0.05^{b}
**4**	44.01±0.05^{d}	ND	ND	13.00±0.37^{d}	22.56±0.66^{ab}	104.62±0.13^{cde}	451.33±23.74^{c}	347.84±3.08^{a}	202.51±2.01^{b}	9.02±0.10^{abc}	22.47±0.05^{b}	25.59±1.10^{b}
**5**	ND	ND	ND	5.46±0.03^{a}	25.80±0.66^{cd}	109.62±1.71^{e}	537.26±2.74^{de}	2102.29±22.48^{g}	256.79±0.77^{e}	ND	86.09±1.21^{d}	ND
**6**	ND	ND	ND	12.98±0.08^{d}	24.85±0.30^{bcd}	106.43±1.00^{cde}	586.05±6.03^{f}	2051.74±2.27^{f}	225.65±1.27^{c}	8.99±0.13^{ab}	141.99±2.62^{g}	ND
**7**	ND	ND	ND	13.86±0.08^{f}	20.29±0.08^{a}	101.55±4.71^{bcd}	308.84±16.28^{b}	357.92±15.52^{a}	203.04±1.10^{b}	9.32±0.01^{c}	22.27±0.30^{b}	24.32±0.11^{a}
**8**	ND	ND	ND	13.93±0.08^{f}	27.01±1.52^{cd}	108.65±0.66^{de}	562.60±0.83^{ef}	2186.08±2.06^{i}	257.08±3.03^{e}	ND	128.71±0.86^{f}	ND
**9**	ND	ND	ND	13.36±0.03^{e}	26.69±2.08^{cd}	102.48±4.21^{bcde}	578.00±0.15^{f}	2091.16±24.55^{g}	256.65±15.98^{e}	8.99±0.42^{ab}	136.23±0.59^{fg}	ND
**10**	44.28±0.44^{d}	62.82±7.6^{a}	23.09±5.6^{a}	13.42±0.15^{e}	24.93±0.18^{bcd}	99.22±0.76^{abc}	707.20±13.17^{h}	1391.69±0.23^{d}	220.56±3.30^{c}	9.07±0.06^{bc}	128.83±6.07^{f}	ND
**11**	ND	ND	ND	5.74±0.05^{a}	27.59±0.83^{c}	121.16±9.1^{f}	526.53±10.69^{d}	2134.84±3.12^{h}	266.88±8.44^{e}	ND	83.68±0.42^{d}	ND
**12**	42.75±0.28^{bc}	ND	ND	13.03±0.10^{d}	24.84±0.69^{bcd}	100.60±0.77^{bc}	633.02±7.76^{g}	1365.15±9.49^{c}	223.07±0.59^{c}	8.73±0.16^{a}	117.14±3.28^{e}	ND

Note: DM: Dry Mass; ClA: Chlorogenic acid; PrA: Protocatechuic acid; Ct: Catechin; CoA: Coumaric acid; CiA: Cinnamic acid; Rt: Rutin; Q+L: Quercetin + Luteolin; Hp: Hesperidin; Kp: Kaempferol; Vn: Vanillin; Nhp: Neohesperidin; Ng: Naringenin; ND = Not detected. Values are means ± SD (n = 3).

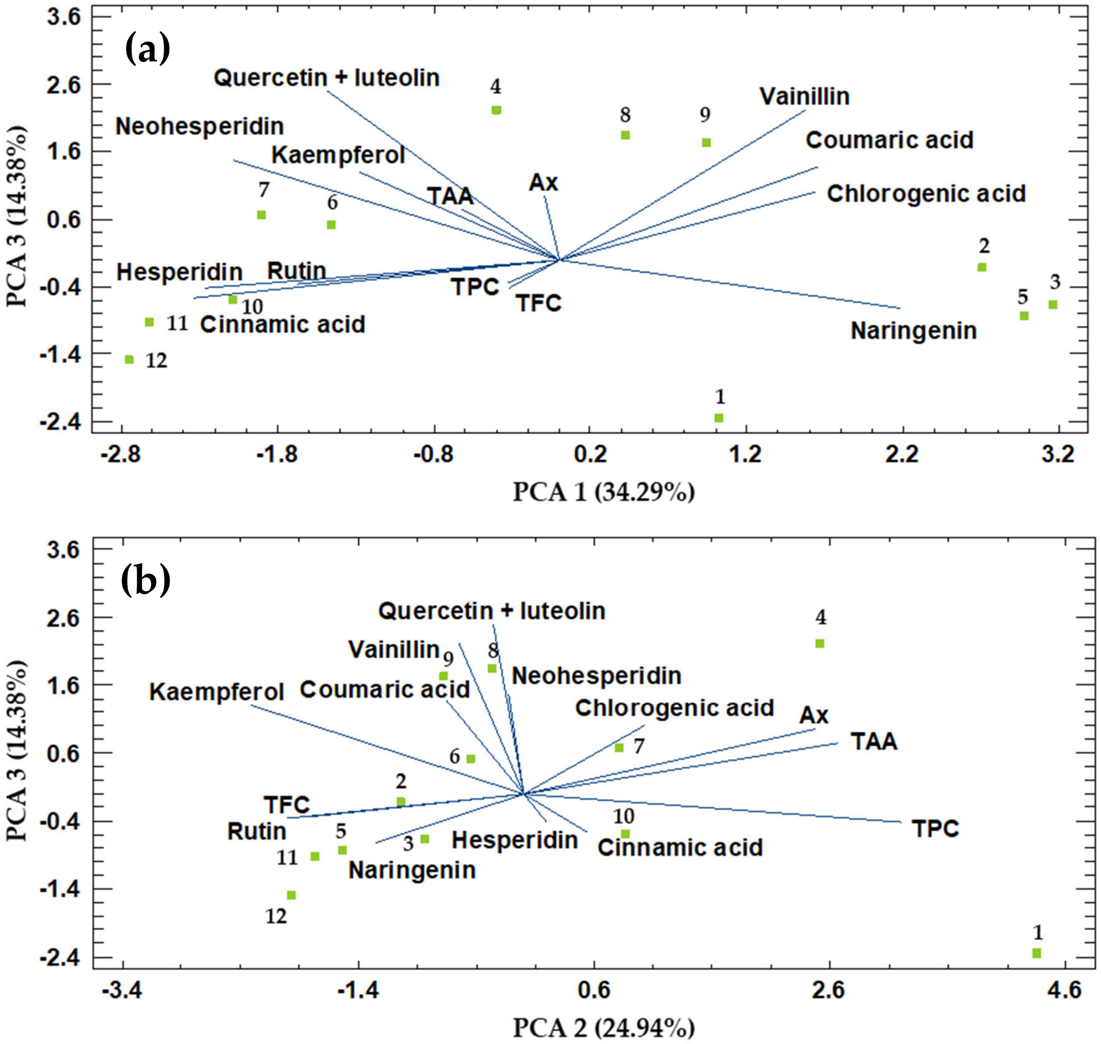

Figure S2: Component analysis (PCA) of the 3×2×2 experimental design for the evaluation of polyphenol extraction from *Citrus aurantium by-products* using different NADES. (a) PCA1 vs. PCA3 and (b) PCA2 vs. PCA3 Numbers are conditions of experiments (Table S1).

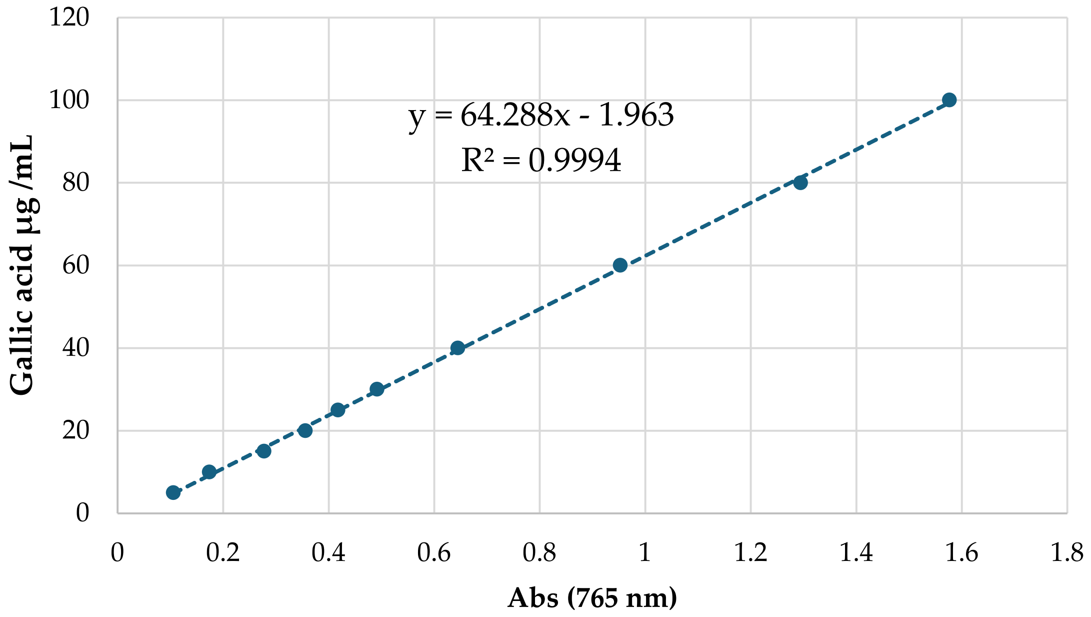

Figure S3: Calibration curve of gallic acid for the determination of total polyphenols from *C. aurantium* by-products.

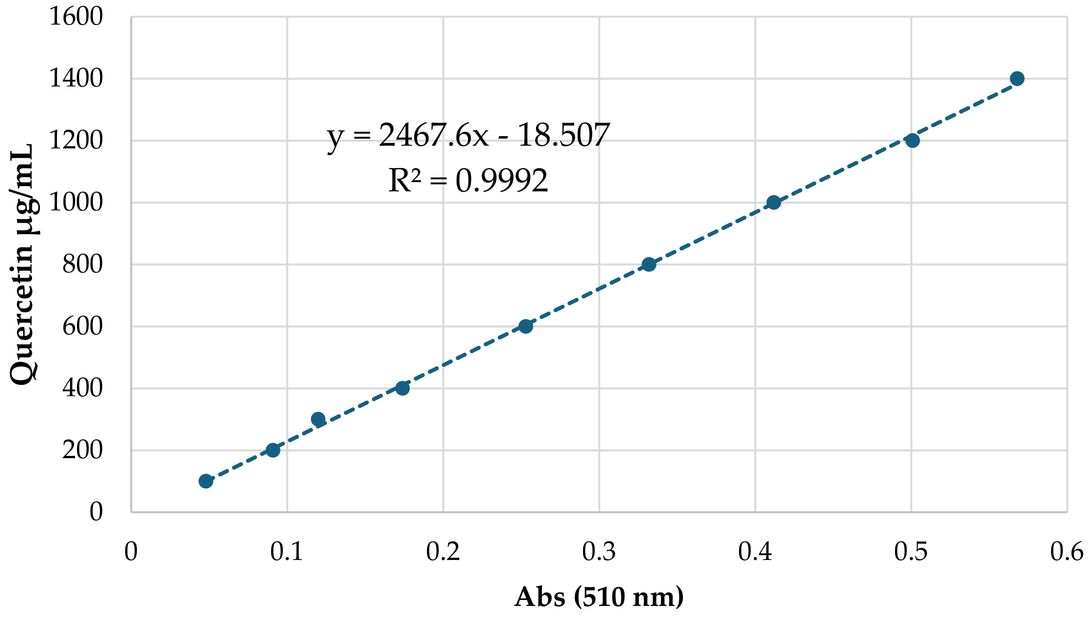

Figure S4: Calibration curve of quercetin for the determination of total polyphenols from *C. aurantium* by-products.

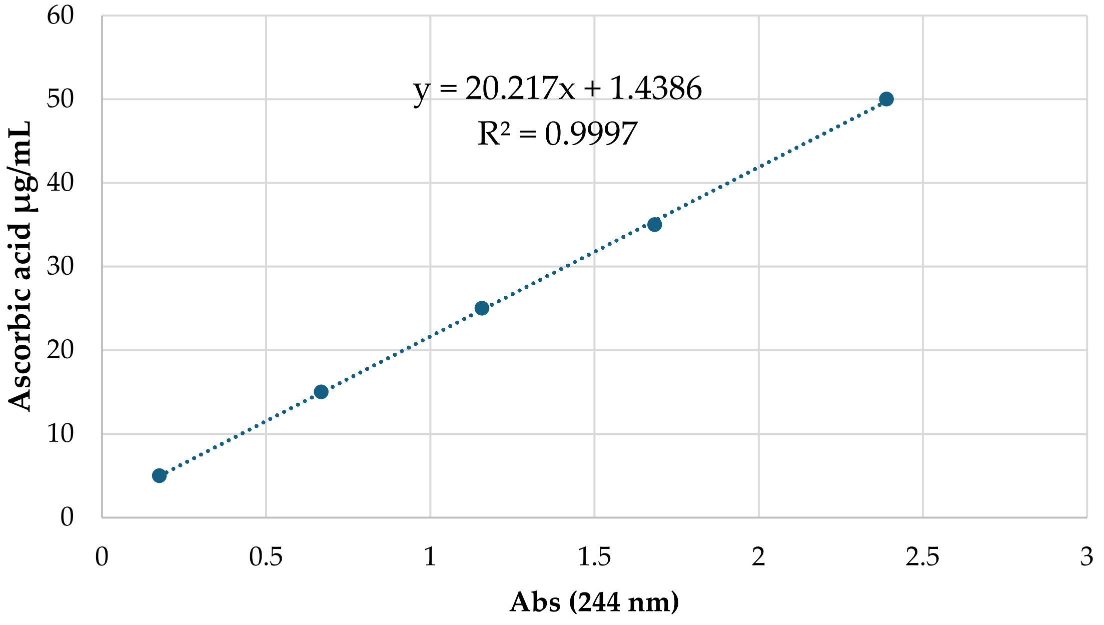

Figure S5: Calibration curve of ascorbic acid for the determination of total polyphenols from *C. aurantium* by-products.
